# Extracellular vesicles of human diabetic retinopathy retinal tissue and urine of diabetic retinopathy patients are enriched for the junction plakoglo bin protein

**DOI:** 10.3389/fendo.2022.1077644

**Published:** 2023-01-06

**Authors:** Jason Mighty, Alfonso Rubio-Navarro, Cui Shi, Jing Zhou, Miguel Flores-Bellver, Søren Heissel, Onyekwere Onwumere, Linda Einbond, Rajendra Gharbaran, Daniel S. Casper, Alberto Benito-Martin, Stephen Redenti

**Affiliations:** ^1^ Lehman College, City University of New York, Bronx, NY, United States; ^2^ Biology Doctoral Program, The Graduate School and University Center, City University of New York, New York, NY, United States; ^3^ Weill Center for Metabolic Health, Cardiovascular Research Institute, Division of Cardiology, Department of Medicine, Weill Cornell Medicine, New York, NY, United States; ^4^ Instituto de Investigación Biosanitaria ibs GRANADA, University Hospitals of Granada-University of Granada, Granada, Spain; ^5^ Excellence Research Unit “Modeling Nature” (MNat), University of Granada, Granada, Spain; ^6^ CellSight Ocular Stem Cell and Regeneration Program, Department of Ophthalmology, Sue Anschutz- Rodgers Eye Center, University of Colorado, Aurora, CO, United States; ^7^ Proteomics Resource Center, The Rockefeller University, New York, NY, United States; ^8^ Department of Ophthalmology, Columbia University Vagelos College of Physicians & Surgeons Naomi Berrie Diabetes Center, New York, NY, United States; ^9^ Universidad Alfonso X El Sabio, Facultad de Medicina. Unidad de Investigación Biomédica, Madrid, Spain

**Keywords:** extracellular vesicle (EV), diabetic retinopathy, JUP, proteomics & bioinformatics, retina, urinary EVs

## Abstract

**Introduction:**

Diabetic Retinopathy (DR) is a potentially blinding retinal disorder that develops through the pathogenesis of diabetes. The lack of disease predictors implies a poor prognosis with frequent irreversible retinal damage and vision loss. Extracellular Vesicles (EVs) present a novel opportunity for pre-symptomatic disease diagnosis and prognosis, both severely limited in DR. All biological fluids contain EVs, which are currently being studied as disease biomarkers. EV proteins derived from urine have emerged as potential noninvasive biomarkers.

**Methods:**

In this study, we isolated EVs from DR retinal tissue explants and from DR patients’ urine, and characterized the vesicles, finding differences in particle number and size. Next, we performed proteomic analysis on human explanted DR retinal tissue conditioned media, DR retinal EVs and DR urinary EVs and compared to normal human retinal tissue, retinal EVs, and urinary EVs, respectively

**Results:**

Our system biology analysis of DR tissue and EV expression profiles revealed biological pathways related to cell-to-cell junctions, vesicle biology, and degranulation processes. Junction Plakoglobin (JUP), detected in DR tissue-derived EVs and DR urinary EVs, but not in controls, was revealed to be a central node in many identified pathogenic pathways. Proteomic results were validated by western blot. Urinary EVs obtained from healthy donors and diabetic patient without DR did not contain JUP.

**Conclusion:**

The absence of JUP in healthy urinary EVs provide the basis for development of a novel Diabetic Retinopathy biomarker, potentially facilitating diagnosis.

## Introduction

Diabetic Retinopathy (DR) is a potentially blinding retinal neurodegenerative and microvascular disorder that develops through the pathogenesis of both type 1 and type 2 diabetes (1). DR currently affects approximately 120 million people worldwide and its prevalence is expected to reach 130 million by 2030 (2). DR is the most frequent cause of new blindness in adults between ages of 20-74. Most patients with type 1 diabetes develop DR within two decades of disease progression (3). In type 2 diabetes, 42.9% of patients with more than 15 years of disease onset will develop DR. DR can be classified into two groups: non-proliferative diabetic retinopathy (NPDR) and proliferative diabetic retinopathy (PDR). DR’s early stages are typically asymptomatic. If advanced DR goes undetected and untreated, irreversible retinal damage and vision loss frequently occur (1) Currently, diagnosis is determined by ophthalmic examination detecting pathologic changes in the neural retina, retinal vasculature, and vitreous (4). DR pathogenesis involves increased blood glucose levels, glutamate toxicity, vascular leakiness, vitreous seeding, and neural retinal cell death. A primary risk factor for DR is uncontrolled levels of blood sugar, due to a lack of insulin, or impaired insulin sensitivity leading to hyperglycemia (5). Complications of diabetes, including hyperglycemia and hyperlipidemia, create a state of chronic inflammation, activating stress pathways in ocular tissue and contributing to DR onset and progression. In pre- and early-DR conditions, retinal tissue exhibits increased metabolic demand, high levels of metabolic waste production and inflammation (6). Extracellular vesicles (EVs) contain specific sets of lipids, proteins, DNAs, and RNAs and have been rendered important for physiological functions as well for diagnostic and therapeutic applications (7). EVs are released by all CNS cell types studied, including neurons, glia, and endothelial cells in both health and disease conditions (8). Interestingly, EVs derived from neural tissue are studied as biomarkers of disease diagnosis, prognosis, and response to treatment (9). EVs have been isolated from most biological fluids including saliva, blood, urine, vitreous and cerebral spinal fluid (10). Furthermore, EVs released from neural tissue can circulate systemically, carrying genetic and molecular signatures of the releasing cells (11). The systemic effects of EVs are a consequence of their molecular cargo which can induce several biological responses in target cells. During the last decade, EVs have been characterized from neurodegenerative diseases (9), cancers (12), metastatic disease (13), allergy (14), and immune system disorders (15). EV proteins derived from urine have emerged as potential noninvasive CNS disease biomarkers (16). Recent studies reveal that human brain EVs contain 1088 proteins, neural retinal EVs contain 1039 proteins, and retinal progenitor EVs contain 1892 protein species (17–19). Our study focuses on identifying an EV-based biomarker for the detection of DR in diabetic patients. First, we analyzed the specific protein cargo of EVs released from *ex vivo* retinal tissue obtained from non-DR and DR donors. Currently, diabetic patient urine is recurrently sampled to monitor ketones, glucose, and progression in nephropathy, including the implementation of urinary EVs in diagnosis (20), pointing it out as a highly desirable biospecimen for biomarker analysis. From this perspective, we measured the specific proteins carried by urine EVs collected from diabetic patients with- and without-DR. We have identified junction plakoglobin as a specific protein cargo of EVs released from both *ex vivo* human DR retinal tissue and human DR urinary EVs, providing the basis for the development of a novel biomarker to diagnose and prevent blindness caused by DR.

## Materials and methods

### Human retinal isolation, culture, and patient samples

All experiments were approved by and performed in compliance with the City University of New York, Lehman College (IRB). Cadaver whole eyes were obtained through the New York Eye-Bank for Sight Restoration or the National Disease Research Interchange. Anterior segments were cut away at the ora serrata, and vitreous and RPE removed from neural retina. Neural retinas were placed in 6-well plates in DMEM plus 10% exosome free fetal bovine serum (FBS), 1% penicillin streptomycin and 0.2% nystatin and cultured at 37°C. Retina conditioned media and retina were collected after five days for analysis. Urine samples were collected after patient consent, stored, and treated as previously described (21). Patient characteristics and application are depicted in **Table 1** (human retina samples) and **Table 2** (human urine samples).

**Table 1:** Human retina patients’ samples information, including gender, age, diabetes (Yes or No), cause of death, application of the samples, time elapsed since donor death and retina culture, eye complications status and comments regarding the presence/absence of diagnosed diabetic retinopathy.

Age	Gender	Diabetes	Ethnicity	Cause of death	Application	Time elapsed before culture (h)	Eye complication Status	Diabetes status and other diagnosis
64	Male	YES	Unknown	Heart failure	Retina and retina EVs proteomic analysis and WB	32	Diabetic Retinopathy	DM Type I
63	Male	YES	Unknown	Renal failure	Retina and retina EVs proteomic analysis and WB	24	Diabetic Retinopathy	DM Type I
54	Male	YES	Hispanic	Coronary Artery Disease	Retina EVs isolation for NTA, Uptake and TEM	22	No Diabetic Retinopathy	Morbidly obese. DM Type II
73	Male	YES	Caucasian	Hypoglicemia	Retina EVs isolation for NTA, Uptake and WB	21	Diabetic Retinopathy	Cataracts, Possible Glaucoma
71	Male	YES	Unknown	Pancreatic cancer	Retina EVs isolation for NTA	24	Diabetic Retinopathy	Cataracts
68	Male	YES	Hispanic	unknown	Retina EVs isolation for NTA and WB	16	No Diabetic Retinopathy	Cataracts, Lasik
68	Male	YES	Hispanic	Cardiopulmonary collapse	Retina EVs isolation for NTA and WB	15	No Diabetic Retinopathy	Cataracts, Lasik
66	Female	YES	Hispanic	unknown	Retina EVs isolation for NTA and WB	22	Diabetic Retinopathy	Cataracts, Lasik
59	Male	YES	Caucasian	Heart disease	Retina EVs isolation for NTA and WB	24	No Diabetic Retinopathy	Hydrocephalus
65,1 ± 5,9						22,2 ± 4,9		
62	Male	NO	Unknown	Heart failure	Retina and retina EVs proteomic analysis and WB	26	Non Diabetic - No DR	Metastatic cancer
56	Male	NO	Unknown	Unknown	Retina and retina EVs proteomic analysis and WB	13	Non Diabetic - No DR	None
26	Male	NO	Caucasian	Heart failure	Retina EVs isolation for NTA and qPCR	40	Non Diabetic - No DR	Drug overdose. No ocular history
27	Male	NO	Unknown	Cholangiocarcinoma stage IV	Retina EVs isolation for WB	12	Non Diabetic - No DR	Artificial tears
35	Male	NO	Causcasian	Endocarditis	Retina EVs isolation for NTA and WB	44	Non Diabetic - No DR	None
21	Male	NO	Caucasian	Lupus	Retina EVs isolation for NTA and WB	20	Non Diabetic - No DR	None
32	Male	NO	Caucasian	Sheptic shock	Retina EVs isolation for NTA	29	Non Diabetic - No DR	None
63	Male	NO	Hispanic	Cardiopulmonary Arrest	Retina EVs isolation for NTA	25	Non Diabetic - No DR	Colorectal cancer
31	Male	NO	Caucasian	Endstage Liver disease	Retina EVs isolation for NTA	48	Non Diabetic - No DR	None
61	Female	NO	Caucasian	Amyotrophic lateral sclerosis	Retina EVs isolation for NTA	36	Non Diabetic - No DR	None
52	Female	NO	Caucasian	Cerebrovascular/Stroke	Retina EVs isolation for NTA	87	Non Diabetic - No DR	None
39,1 ± 16,2						37,8 ± 21,7		

Average ± standard dev. Included below age and time elapsed columns. EVs, Extracellular vesicles; WB, Western Blot; NTA, Nanoparticle tracking analysis; TEM, Transmission electron microscopy; DM, Diabetes Mellitus; DR, Diabetic Retinopathy.

**Table 2:** Human urine patients’ samples information, including gender, age, diabetes (Yes or No), ethnicity, application of the samples, diabetic status, diagnosis and glycated hemoglobin levels (A1C).

Age	Gender	Diabetes	Ethnicity	Application	Diabetic Status	Diagnosis	A1C (%)
75	Male	YES	Caucasian	Fundus No DR Fig.3 - Urinary EVs isolation	No Diabetic Retinopathy	DM Type II	6.2
51	Male	YES	Caucasian	Fundus DR Fig.3 - Urinary EVs isolation	Severe NPDR	DM Type I	10.2
65	Male	YES	Caucasian	Urinary EVs Isolation	Severe NRPD	DM Type I	8.4
38	Male	YES	Caucasian	Urinary EVs Isolation, TEM, qPCR	Severe NRPD	DM Type I	8.2
42	Male	YES	Caucasian	Urinary EVs Isolation	No Diabetic Retinopathy	DM Type I	8.6
34	Female	YES	Hispanic	Urinary EVs Isolation	No Diabetic Retinopathy	DM Type I	7.1
36	Male	YES	Caucasian	Urinary EV isolation	Mild NRPD	DM Type I	7.8
46	Male	YES	Caucasian	Urinary EVs Isolation	Mild NRPD	DM Type I	8.5
38	Male	YES	Caucasian	Urinary EVs Isolation	Mild NRPD	DM Type I	7.1
48	Male	YES	Unknown	Urinary EVs isolation, NTA and proteomic analysis	Severe NPDR	DM Type I, CSME	10.1
35	Male	YES	Unknown	Urinary EVs isolation, NTA and proteomic analysis	Severe NPDR	DM Type I	8.2
46,2 ± 13,2							
46	Male	NO	Caucasian	Urinary EVs isolation, NTA and proteomic analysis	Non Diabetic	NA	NA
52	Male	NO	Caucasian	Urinary EVs isolation, NTA and proteomic analysis	Non Diabetic	NA	NA

EVs, Extracellular vesicles; NTA, Nanoparticle tracking analysis; DM, Diabetes Mellitus; DR, Diabetic Retinopathy; NPDR, Non-proliferative diabetic retinopathy; CSME, Clinically Significant Macular Edema; NA, Not applicable. Average ± standard dev. included below age column.

### Retinal tissue histology

Retinas were immersion fixed in 4% paraformaldehyde, rinsed three times in PBS, and cryoprotected first in 10%, then in 30% sucrose for 12 h each at 4°C. Retinas were then placed in cryomolds containing optimum cutting temperature compound (ProSciTech), frozen on dry ice and cryo-sectioned at 20 μm. Retinal slices were adhered to glass slides, images obtained and lamina thickness analyzed using a Nikon Ti microscope with a 20X objective, and phase and DAPI nuclear labeling (358/461).

### Human retinal EVs isolation and characterization

Conditioned media was collected from 6-well plates and EVs isolated using ultracentrifugation as previously described (17). EVs were suspended in phosphate-buffered saline (PBS) and stored at −80 °C for analysis. Diameter and concentration were assessed using the NanoSight NS500 system. Nanoparticle Tracking Analysis (NTA) software 2.3 was used to track trajectories and diameters of nanoparticles. Results are displayed as frequency sized distribution graphs describing the number of particles per milliliter.

### Transmission electron microscopy and immunogold labelling

EVs fixed with 2% paraformaldehyde were placed on glow discharged formvar-carbon coated nickel grids. Grids were blocked for 10 min with 1% fish skin gelatin (Sigma-Aldrich). Primary antibodies (Tsg101, CD63; Abcam, UK) were suspended in blocking solution and applied for 2h. Nanogold-labeled Fab’ anti-rabbit or anti-mouse (Nanoprobes, NY), or 5nm, 10nm gold conjugated goat anti-mouse antibodies used as control (Ted 31 Pella Inc. Redding, CA) were applied in the correlated antibody incubation buffer for 1 hour. The grids were then washed with PBS and fixed in 1% glutaraldehyde for 5 min. After washing, the grids were either placed directly into methylcellulose for 5 nm or 10 nm gold embedding or allowed to continue with silver enhancement of nanogold using HQ Silver enhancement kit (Nanoprobes, NY). Finally, grids were embedded in a mixture of 3% uranyl acetate and 2% methylcellulose. Stained grids were examined under Philips CM-12 electron microscope and photographed with a Gatan (1kÅ~1k) digital camera.

### Retinal RNA labeling and EV RNA cargo transfer analysis

Using the culture and isolation methods described above, the RNA-specific dye SytoRNA Select (Invitrogen) was used on whole human retina with minor modifications to the manufacturer’s protocol. Retinas were placed in a 2.5uM solution of dye and incubated at 37°C, 5% CO_2_ for one hour, and then rinsed three times with fresh media. SytoRNA labeled retina were then incubated in the TRITC fluorescent lipophilic dye PKH26 (Sigma) according to the manufacturer’s instructions. Retina was placed in 500uL diluent and transferred to a 4 ×10^-6^ M dye solution and incubated at room temperature for 15 minutes on an orbital shaker.

### Transwell EVs diffusion culture

Human retinas were collected as described above, lightly dissociated by trituration, and cultured in 12 well glass bottom plates (MatTek corporation). Whole retina stained with SytoRNA Select (Invitrogen) and PKH26 (Sigma), as described above, were cultured in Transwell inserts with 0.4μm pore PET membranes above non-labeled dissociated retina and co-cultured for five days. Double SytoRNA (green) RNA and PKH26 (red) lipid-labelled EVs released by adult retina diffused through the transwell membrane pores and were imaged contacting cells on glass-bottom wells.

### Super resolution imaging of retinal cells containing transferred EVs

Double-labeled EVs were fixed with 4% PFA and mounted with DAPI Prolong Gold mounting media for imaging. Multichannel structured illumination microscopy (SIM) images were acquired using a Nikon Structured Illumination N-SIM system on an inverted Nikon ECLIPSE Ti-E. Z stack images were acquired using the electron-multiplying CCD cameras (Andor iXon3 DU897). Three reconstruction parameters (Illumination Modulation Contrast, High-Resolution Noise Suppression, and Out of Focus Blur Suppression) were extensively tested to generate consistent images. The acquired images were then processed using Nikon Elements software. 3D reconstruction was generated using Imaris software (Bitplane).

### RNA and protein isolation and western blot analysis

Total RNA was extracted and purified from EV pellets using the Trizol reagent according to the manufacturer’s protocol (Thermo Fisher). Protein was isolated from retinal tissue using Pierce lysis buffer (Thermo Fisher) with 1% protease inhibitor and 1% phosphatase inhibitor. For EV protein isolation, centrifuged and pelleted EVs were suspended in 200ul PBS. Protein concentrations were obtained using the bicinchoninic acid assay (BCA) assay (Thermo Fisher). Primary antibodies were mouse anti-γ-catenin (Santa Cruz, sc-33634, 1:1000), rabbit anti-actin (Cell Signaling, #8456, 1:5,000), mouse anti-CD81 (Santa Cruz, sc-166028, 1:1000), rabbit anti-CD63 (Novus, nbp2-67425, 1:1000). Blots were developed with the chemiluminescence method (ECL Luminata Crescendo, WBLUR0500, Millipore). Band density was quantified using Fiji/ImageJ (NIH).

### Mass spectrometry

Whole cell lysate and EV-enriched samples from human retinas were denatured in 8M urea, reduced with 10mM DTT, and alkylated with 50mM iodoacetamide. This was followed by proteolytic digestion with endoproteinase LysC (Wako Chemicals) overnight, and with trypsin (Promega) for 6h at room temperature. Samples were dried and resolubilized in 2% acetonitrile and 2% formic acid. Samples were injected for analysis by reversed phase nano-LC-MS/MS (Ultimate 3000 coupled to a QExactive Plus, Thermo Scientific). After loading on a C18 trap column (PepMap, 5μm particles, 100μm x 2cm, Thermo Scientific) peptides were separated using a 12cm x 75μm C18 column (3μm particles, Nikkyo Technos Co., Ltd. Japan) at a flow rate of 200nL/min, with a gradient increasing from 5% BufferB (0.1% formic acid in acetonitrile)/95% Buffer A (0.1% formic acid) to 40% Buffer B/60%Buffer A, over 140 minutes. All LC-MS/MS experiments were performed in data dependent mode with lock mass of m/z 445.1200345.

### Protein profiling and bioinformatic analysis

Mass spectrometry data were searched against a human database using MaxQuant (version 1.5.0.30 45). Oxidation of methionine and N-terminal protein acetylation were allowed as variable modifications, while all cysteines were treated as being carbamidomethylated. Precursor mass tolerance was set at 4.5ppm while a 20ppm tolerance was allowed for fragment ions. Two missed cleavages were allowed for specific tryptic search. The “match between runs” option was enabled. False discovery rates at the protein and peptide level were set to 1%. For system biology analysis we used IPA software, which allows for functional enrichment, core analysis and other features (Qiagen) (22). The mass spectrometry proteomics data have been deposited to the ProteomeXchange Consortium *via* the PRIDE partner repository with the dataset identifier PXD034626

### Statistical analysis

Error bars in the graphical data represent mean ± S.E.M. unless specified. For *in vivo* and *in vitro* assays, specifics regarding the number of independent experiments and biological replicates are stated in the figure legends except when only one experiment was performed. When appropriate, statistical significance was determined by applying two-tailed unpaired or paired Student’s t-tests using GraphPad Prism software v.9.1.0.

## Results

### Characterization of EVs from diabetic retina

**Figure 1:**
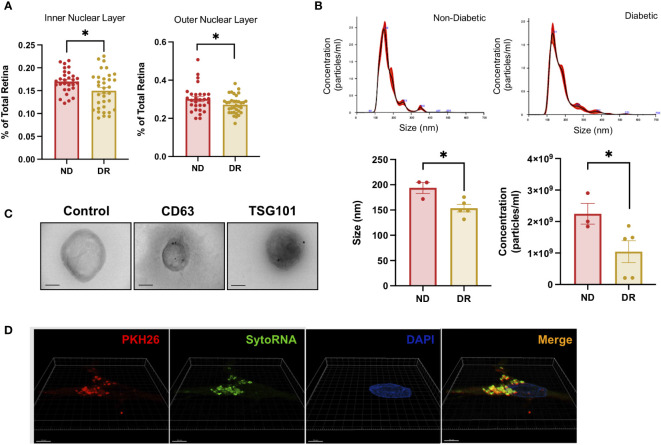
Diabetic Retinopathy and Non-Diabetic Human Retina EV release rate, characterization, and genetic transfer. **(A)** Retinal outer nuclear layer (ONL) and inner nuclear layer (INL) percent thickness were compared between DR and non-Diabetic retina *ex vivo*. Mean ± S.E.M.; *p< 0.05 Student´s t-test. **(B)** Particle size distribution in non-diabetic and diabetic retina (upper panels) and concentration and size (lower panels) were measured in triplicates. Mean ± S.E.M.; *p < 0.05 Student´s t-test. **(C)** TEM imaging of EVs isolated from human diabetic retina conditioned media from left to right Negative stain control, CD63 and TSG 101 staining. Scale Bar= 100nm. **(D)** Representative images of Lipid membrane and RNA labeled EVs uptake by unlabeled retinal cells. Super-resolution microscopy was used to visualize RNA (SytoRNA/green) and lipid membrane (PKH26/red) labeled EVs released from adult retina and taken up by control non-labeled adult retinal cells in a transfer experiment.

During diabetes, microvascular complications lead to tissue ischemia resulting in a thickness reduction of the neural retina (23) as shown in a comparison of normal and DR *ex vivo* retinas used in this study (**Figure 1A**). We first characterize EVs released from *ex vivo* DR and non-diabetic human retinal tissue (patient samples characteristics included in **Table 1**). We isolated EVs from day 5 human retina-conditioned media. Times elapsed since patients’ deceases and retina culture are included in **Table 1** for all samples used in this study. We used nanoparticle tracking analysis technology (NTA) to determine their concentration and size and found a decrease in both parameters in diabetic retina derived-EVs when compared with non-diabetic retinal EVs (**Figure 1B**). In average, EVs size and cell count were bigger in non-diabetic retina EVs. Immunogold detection confirmed the proper localization of canonical EVs proteins including CD63 and TSG101 at the surface of isolated retinal EVs (**Figure 1C**). Since EVs have shown a potential biological relevance, we evaluated functional integrity of the molecular cargo of our EVs using an RNA labelling transfer assay (**Figure 1D**). The SYTO RNA Select is a permeant nucleic acid stain selective for RNA, with less potential non-specific labeling when compared with lipophillic dyes (24, 25). Super-resolution microscopy revealed internalization of labeled EVs by non-labeled dissociated retinal neurons, localizing mainly to the cytoplasm (**Figure 1D**), demonstrating cell-to-cell cargo transfer by EVs released from adult human retinas.

### Comparison between DR and non-diabetic human EVs and retinal tissue proteomes

**Figure 2:**
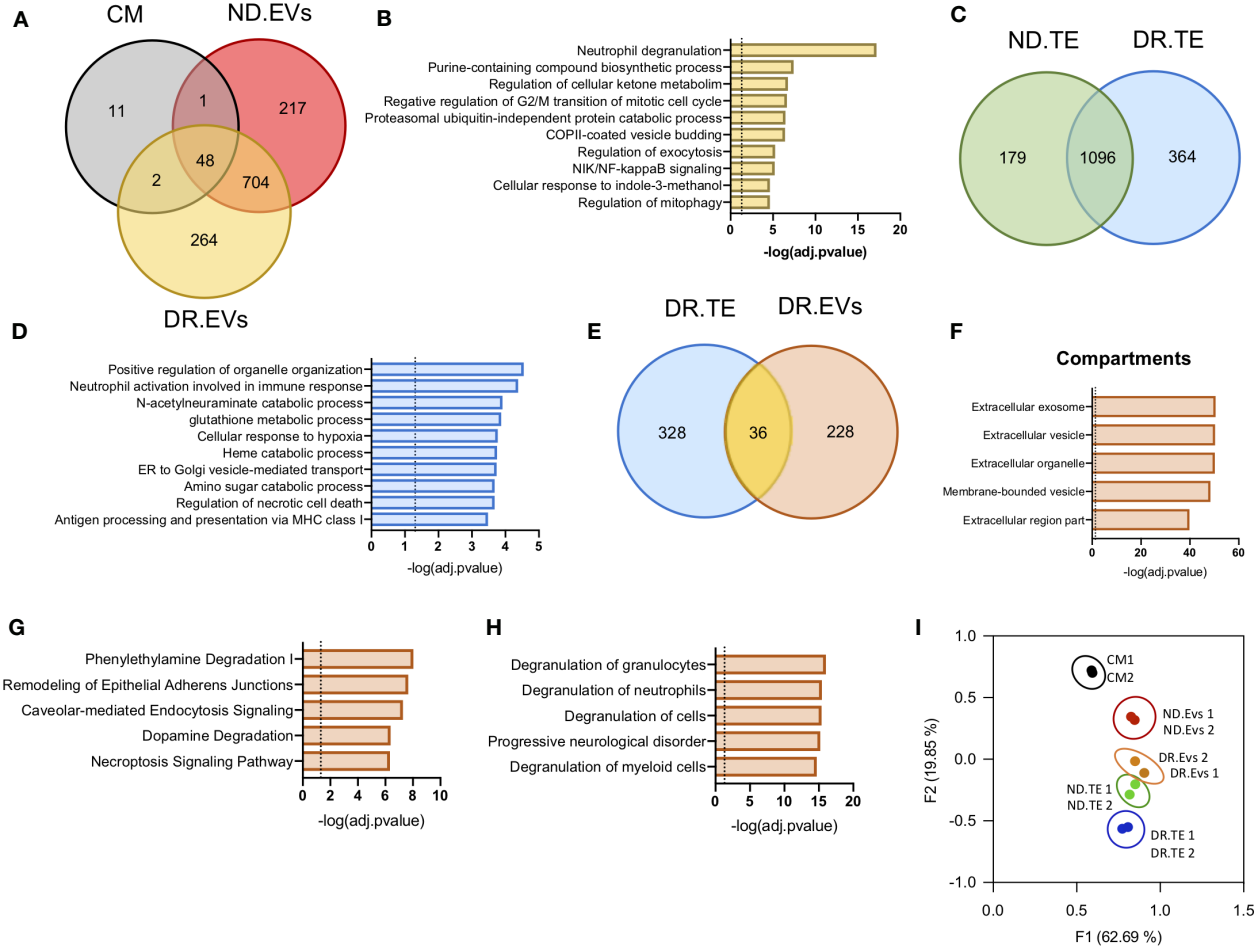
Proteomic analysis of EVs released by Diabetic Retinopathy and Non-Diabetic human retinal tissue. **(A)** Venn diagrams showing protein data set intersections between Control Media (CM) and retinal EVs from Diabetic Retinopathy (DR.EVs) and Non-Diabetic (ND.EVs) patients. **(B)** Pathway enrichment analysis of exclusive proteins in DR.EVs. **(C)** Venn diagrams showing shared proteins between retinal tissue explants of non-diabetic (ND.TE) and diabetic patients (DR.TE). **(D)** Pathway enrichment analysis of exclusive proteins in DR.TE. **(E)** Venn diagrams of exclusive proteins detected in DR.EVs and in DR.TE. **(F)** Prediction analysis to determine exclusive DR.EVs protein subcellular localization. Pathway **(G)** and biological process **(H)** enrichment analysis of exclusive proteins in DR.EVs. **(I)** PCA scatter plot comparing the proteomes of Control Media (CM), ND.EVs, DR.EVs, ND.TE and DR.TE.

To characterize the specific protein cargo in EVs released from human retinal tissue, we performed proteomic analysis of EVs released from DR (DR.EVs) or non-diabetic (ND.EVs) human retinal tissue *ex vivo*. Human retinal EVs contained between 1500 and 2000 proteins, 264 being exclusive to DR EVs (**Figure 2A** and **Supplementary Table 1**). To gain insight into the molecular signaling associated with DR.EVs, we performed pathway analysis on the 264 exclusive proteins present in DR.EVs. The DR.EV specific proteins correlated to inflammatory signaling, purine metabolism, and ketone metabolism, known pathways in the pathogenesis of diabetes (**Figure 2B** ) (6, 26, 27). Next, we analyzed the proteome of whole retinas from diabetic (DR.TE) and non-diabetic tissue explants (ND.TE), finding 364 proteins exclusively in DR tissue explants (**Figure 2C** and **Supplementary Table 2**). The DR.TE exclusive proteins correlate to pathogenic signaling in diabetes including inflammation, oxidative stress, hypoxia, necrotic cell death, and sugar metabolism (**Figure 2D**). Next, we compared proteins detected only in DR tissue explants with proteins detected exclusively in DR extracellular vesicles to discard soluble factors and potential protein contaminants isolated with EVs (**Figure 2E**). Next, we classified EV-exclusive proteins as either secreted, soluble, or EV-contained peptides (**Figure 2F**). Proteomic analysis revealed 228 proteins exclusively detected in DR extracellular vesicles that were further correlated to compartments *via* bioinformatics (**Supplementary Table 3**). Pathway analysis revealed that DR.EVs were enriched for proteins associated with inflammation; degradation pathways including phenylethylamine and dopamine; remodeling of epithelial adherent junctions; caveolar-mediated endocytosis signaling and necroptosis signaling (**Figure 2G**). Interestingly, top functions associated with the 228 DR.EV-exclusive proteins correlate with immune cell degranulation or progressive neurological disorders (**Figure 2H**). Principal component analysis (PCA) was applied to show correlations of functionally related datasets and revealed that proteomic profiles were different between diabetic and non-diabetic retinal tissue, DR.EVs and non-DR.EVs (**Figure 2I**).

### Human diabetic retinopathy patients urinary EV characterization

**Figure 3:**
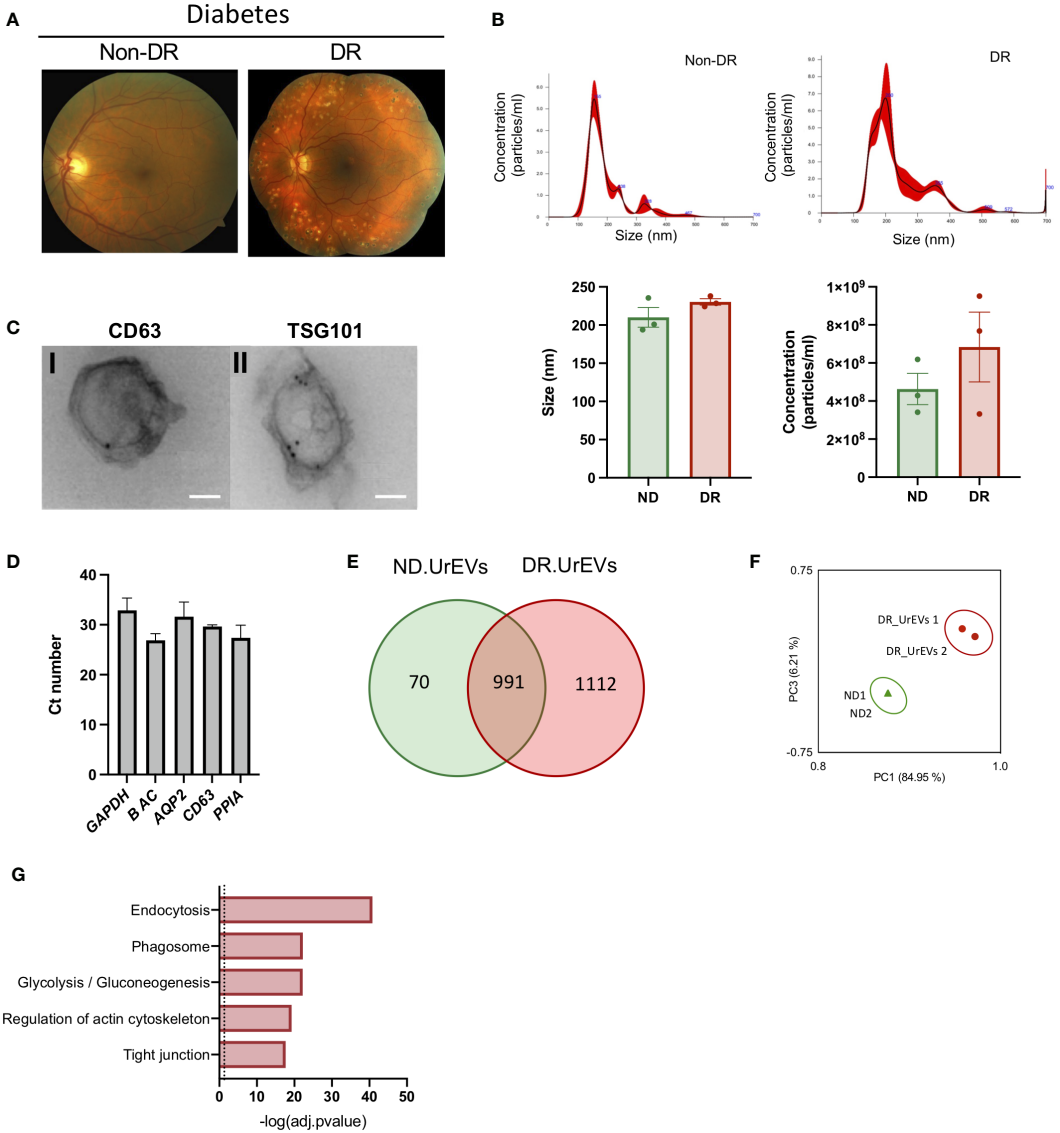
Human urine EVs from diabetic patients contain canonical EV markers and genetic cargo. **(A)** A) Representative Fundus images of Diabetic No DR (left panel) and Diabetic moderate non-proliferative diabetic retinopathy. (Right panel). **(B)** Representative nanoparticle tracking analysis of urinary EVs (upper panel). Average size and concentration of urinary EVs released from Non-DR and DR patients (bottom panel). Size Student’s t-test, p = 0.211, concentration Student’s t-test, p = 0.3341 **(C)** Immunogold representative TEM imaging of EVs isolated from human urine, labeled for CD63 (left panel) and TSG 101 (right panel). Scale: 100nm. **(D)** qPCR analysis reveals the presence of CD63 and urinary markers AQP2 and Cyclophillin A in Urinary EVs. **(E)** Venn diagrams showing shared proteins between ND.UrEVs and DR.UrEVs. **(F)** PCA scatter plot comparing the proteomes of ND.UrEVs and DR.UrEVs. **(G)** Pathway enrichment analysis of exclusive proteins in DR.UrEVs.

DR pathogenesis involves observable changes in retinal microvasculature and retinal neural density. Fundus images comparing normal and affected retina currently provide the basis for DR diagnosis (**Figure 3A**). EVs have become valuable diagnostic and prognostic non-invasive disease biomarkers in scenarios in which pathology is not easily detectable (12). Thus, we decided to study how DR modulated urinary EVs proteomic profiles. We first characterized urinary EVs from diabetic patients with DR (DR.UrEVs) or without DR (ND.UrEVs). Patient´s characteristics are showed in **Table 2**, including A1C levels. NTA analysis revealed that DR.UrEVs slightly larger than non-DR.UrEVs, and the number of particles/ml is higher in DR.UrEVs, but the results were not statistically significant. (**Figure 3B** top histograms and bottom panels). We assessed morphology and canonical EV protein’s location (CD63 and TSG101) by TEM (**Figure 3C**). We also confirmed the cargo of transcripts for Aquaporin 2, CD63 and cyclophilin A in urinary EVs (**Figure 3D**). Quantitative proteomic analysis of human samples from diabetic patients with DR and human samples without DR urinary EVs showed that 1112 proteins are detected exclusively in DR.UrEVs (**Figure 3E** and **Supplementary Table 4**). Principal Component Analysis (**Figure 3G**) depicts grouped proteomic expression profiles by their pathological status. Functional enrichment analysis showed that DR.UrEVs are enriched for proteins associated to endocytosis, phagosome, glucose metabolism, regulation of actin cytoskeleton, and tight junction (**Figure 3G**), pathways previously described in diabetic pathology (28, 29).

### Integrative analysis of DR tissue explants EVs and urinary DR EVs proteomic profiles

**Figure 4:**
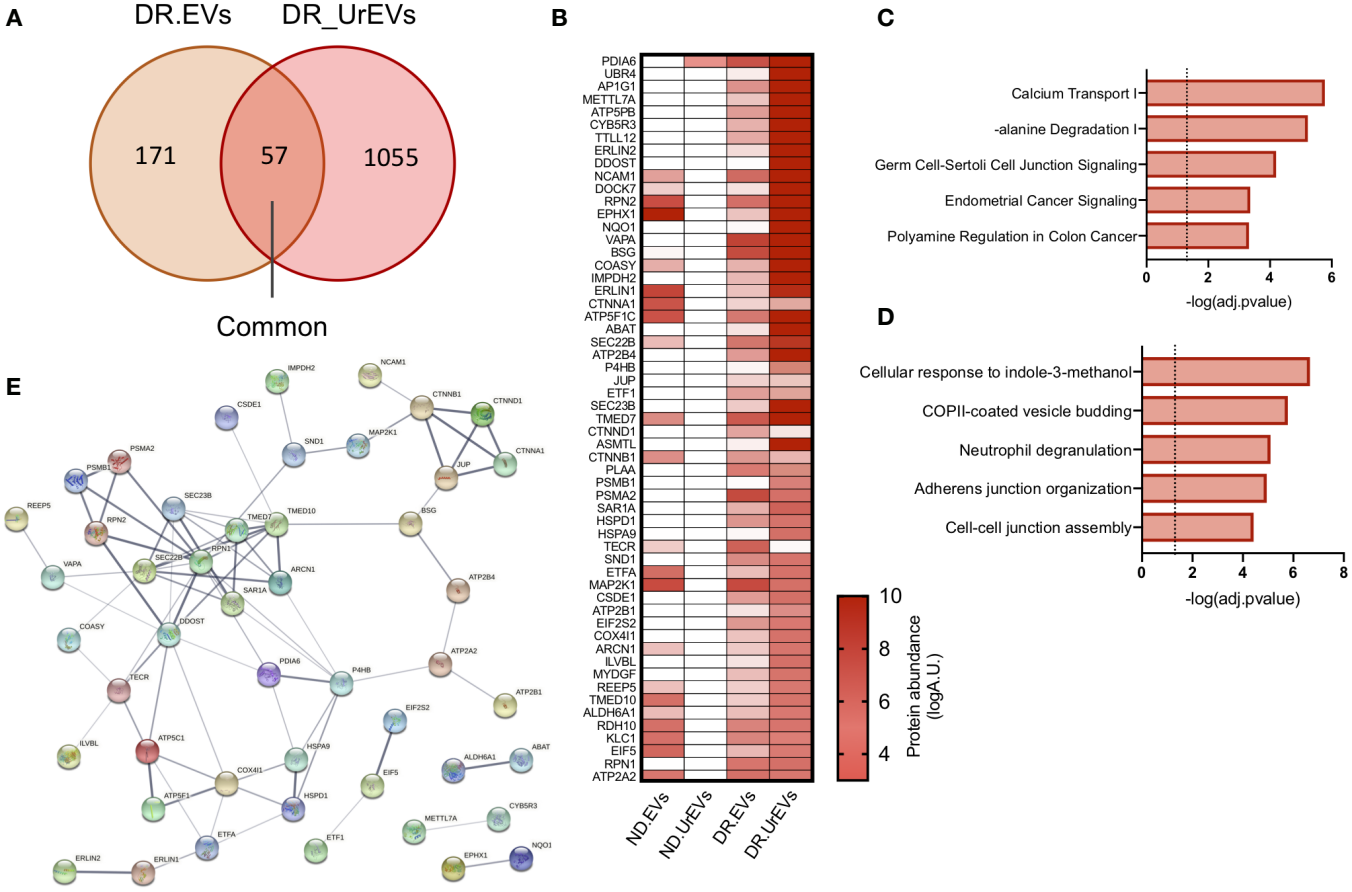
Urine EVs contain proteins observed in retinal EV from DR patients. **(A)** Venn diagrams comparing proteome from DR retina tissue derived EVs and DR Urinary EVs. **(B)** Heatmap of 57 common proteins showing expression in left to right, Nondiabetic retinal tissue derived EVs (ND. EVs), Non diabetic Urinary EVs (ND.UrEVs), Diabetic retinopathy retinal tissue derived EVs (DR.EVs) and Diabetic retinopathy Urinary EVs (DR.UrEVs). Pathway **(C)** and biological process **(D)** enrichment analysis of 57 common proteins found in urinary and retinal EVs from DR patients. **(E)** STRING protein-protein interaction networks of common protein observed in EVs from DR patients.

The passage of circulating EVs into the urine is reported to result from glomeruli filtration deficiencies associated with diabetic kidney damage (30). We evaluate if urinary DR EVs resemble DR tissue explant derived EVs, comparing exclusively detected proteins from both sample types. This comparison revealed 57 common EV proteins (**Figure 4A**; **Table 3**), primarily observed in urinary EVs and retina derived EVs from diabetic patients and not in urinary EVs obtained from healthy non-diabetic samples (**Figure 4B**). Organized in a heatmap, we observed these 57 proteins highly detected in vesicles obtained from DR samples, both urine and retinal tissue, but not in healthy non-diabetic samples from urine or retina tissue explants. Next, we performed pathway and GO term enrichment analysis (**Figures 4C, D**) and protein–protein interaction network analysis (**Figure 4E**). Pathway analysis revealed calcium transport, alanine degradation, polyamine regulation and cell-cell junction (**Figure 4C**). Enrichment analysis presents cell-cell and adherent junction organization, vesicle budding, neutrophil degranulation and cellular response to indole-3-methanol (**Figure 4D**).

**Table 3:** 57 proteins detected both in diabetic retinopathy extracellular vesicles released from human retina tissue and diabetic retinopathy urinary extracellular vesicles.

Gene	Accession	Description	DRUrEVs	NormUrEVs	DR_EVs Avg Int	NoDR_EVs Avg Int
JUP	P14923	Junction plakoglobin	4.9E+08	–	5.3E+08	–
ATP5PB	P06576	ATP synthase subunit beta, mitochondrial	3.4E+08	–	8.3E+08	3.4E+08
ALDH6A1	Q02252	Methylmalonate-semialdehyde dehydrogenase	1.4E+08	–	1.6E+08	–
HSPD1	P10809	60 kDa heat shock protein, mitochondrial	1.2E+08	–	1.2E+08	–
NQO1	B4DLR8	NAD(P)H dehydrogenase [quinone] 1	1.1E+08	–	1.0E+09	–
CTNNB1	B4DGU4	Catenin beta-1	8.8E+07	–	4.6E+08	7.5E+07
P4HB	P07237	Protein disulfide-isomerase	8.1E+07	–	1.1E+09	7.7E+07
BSG	P35613	Basigin	6.4E+07	–	3.5E+08	2.9E+08
METTL7A	H0YI09	Methyltransferase-like protein 7A (Fragment)	5.1E+07	–	8.1E+08	–
ETFA	P13804	Electron transfer flavoprotein subunit alpha, mitochondrial	4.8E+07	–	8.4E+07	–
COX4I1	P13073	Cytochrome c oxidase subunit 4 isoform 1, mitochondrial	4.4E+07	–	7.1E+07	–
ATP5F1	P24539	ATP synthase subunit b, mitochondrial	4.4E+07	–	1.5E+08	–
HSPA9	P38646	Stress-70 protein, mitochondrial	4.3E+07	–	9.9E+07	–
TECR	Q9NZ01	Very-long-chain enoyl-CoA reductase	3.7E+07	–	4.7E+08	1.3E+08
RPN1	P04843	Dolichyl-diphosphooligosaccharide–protein glycosyltransferase 1	3.4E+07	–	1.6E+08	–
CTNND1	C9JZR2	Catenin delta-1	3.3E+07	–	1.6E+08	–
TTLL12	Q14166	Tubulin–tyrosine ligase-like protein 12	3.3E+07	–	1.7E+08	2.4E+08
CYB5R3	P00387	NADH-cytochrome b5 reductase 3	3.0E+07	–	1.3E+09	–
ATP2A2	P16615	Sarcoplasmic/endoplasmic reticulum calcium ATPase 2	2.9E+07	–	2.0E+08	–
DDOST	A0A0C4DGS1	Dolichyl-diphosphooligosaccharide–protein glycosyltransferase	2.9E+07	–	7.7E+07	–
RPN2	P04844	Dolichyl-diphosphooligosaccharide–protein glycosyltransferase 2	2.9E+07	–	1.1E+08	–
TMED10	P49755	Transmembrane emp24 domain-containing protein 10	2.8E+07	–	1.4E+08	1.0E+08
COASY	Q13057	Bifunctional coenzyme A synthase	2.8E+07	–	5.5E+07	–
AP1G1	O43747	AP-1 complex subunit gamma-1	2.6E+07	–	1.2E+07	–
PDIA6	Q15084	Protein disulfide-isomerase A6	2.4E+07	–	6.1E+08	9.1E+07
MAP2K1	Q02750	Dual specificity mitogen-activated protein kinase kinase 1	2.3E+07	–	2.2E+08	2.7E+08
ARCN1	P48444	Coatomer subunit delta	2.0E+07	–	1.1E+08	2.0E+08
IMPDH2	P12268	Inosine-5’-monophosphate dehydrogenase 2	1.9E+07	–	1.3E+08	8.3E+07
PSMB1	P20618	Proteasome subunit beta type-1	1.9E+07	–	2.5E+08	2.4E+08
MYDGF	Q969H8	UPF0556 protein C19orf10	1.8E+07	–	1.4E+08	–
SEC22B	O75396	Vesicle-trafficking protein SEC22b	1.7E+07	–	1.9E+08	–
PSMA2	P25787	Proteasome subunit alpha type-2	1.7E+07	–	6.3E+08	6.3E+08
ATP2B4	P23634	Plasma membrane calcium-transporting ATPase 4	1.6E+07	–	4.1E+08	–
ETF1	B7Z7P8	Eukaryotic peptide chain release factor subunit 1	1.5E+07	–	9.6E+07	1.6E+08
EPHX1	P07099	Epoxide hydrolase 1	1.5E+07	–	2.4E+09	–
SND1	Q7KZF4	Staphylococcal nuclease domain-containing protein 1	1.5E+07	–	3.3E+08	2.4E+08
CTNNA1	P35221	Catenin alpha-1	1.4E+07	–	8.3E+07	–
TMED7	Q8NBU8	Protein TMED7-TICAM2	1.4E+07	–	7.2E+07	5.6E+07
ATP2B1	P20020	Plasma membrane calcium-transporting ATPase 1	1.4E+07	–	3.5E+08	–
ABAT	P80404	4-aminobutyrate aminotransferase	1.2E+07	–	5.0E+07	5.3E+07
VAPA	Q9P0L0	Vesicle-associated membrane protein-associated protein A	1.1E+07	–	2.1E+08	1.1E+08
EIF5	I3L397	Eukaryotic translation initiation factor 5A-1	1.1E+07	–	–	1.3E+08
KLC1	G3V2E7	Kinesin light chain 1	1.0E+07	–	3.0E+07	7.4E+07
RDH10	Q8IZV5	Retinol dehydrogenase 10	1.0E+07	–	3.3E+08	–
ILVBL	A1L0T0	Acetolactate synthase-like protein]	8.9E+06	–	1.2E+08	–
PLAA	E5RIM3	Phospholipase A-2-activating protein	8.4E+06	–	2.2E+07	1.7E+07
ASMTL	O95671	N-acetylserotonin O-methyltransferase-like protein	7.9E+06	–	1.4E+08	1.3E+08
NCAM1	P13591	Neural cell adhesion molecule 1	7.6E+06	–	4.2E+08	–
SAR1A	Q9NR31	GTP-binding protein SAR1a	7.4E+06	–	2.6E+08	–
SEC23B	Q15437	Protein transport protein Sec23B	6.7E+06	–	1.2E+08	2.8E+08
DOCK7	Q96N67-2	Isoform 2 of Dedicator of cytokinesis protein 7	6.7E+06	–	4.9E+06	–
ERLIN1	O75477	Erlin-1	6.5E+06	–	2.5E+08	–
ERLIN2	E5RHW4	Erlin-2 (Fragment)	6.3E+06	–	4.2E+08	–
REEP5	Q00765	Receptor expression-enhancing protein 5	6.2E+06	–	5.2E+08	2.2E+08
CSDE1	O75534	Cold shock domain-containing protein E1	4.9E+06	–	6.5E+07	6.5E+07
UBR4	Q5T4S7	E3 ubiquitin-protein ligase UBR4	2.9E+06	–	1.3E+08	4.6E+07
EIF2S2	P20042	Eukaryotic translation initiation factor 2 subunit 2	2.5E+06	–	4.4E+07	–

### Validation of plakoglobin in urinary DR EVs

**Figure 5:**
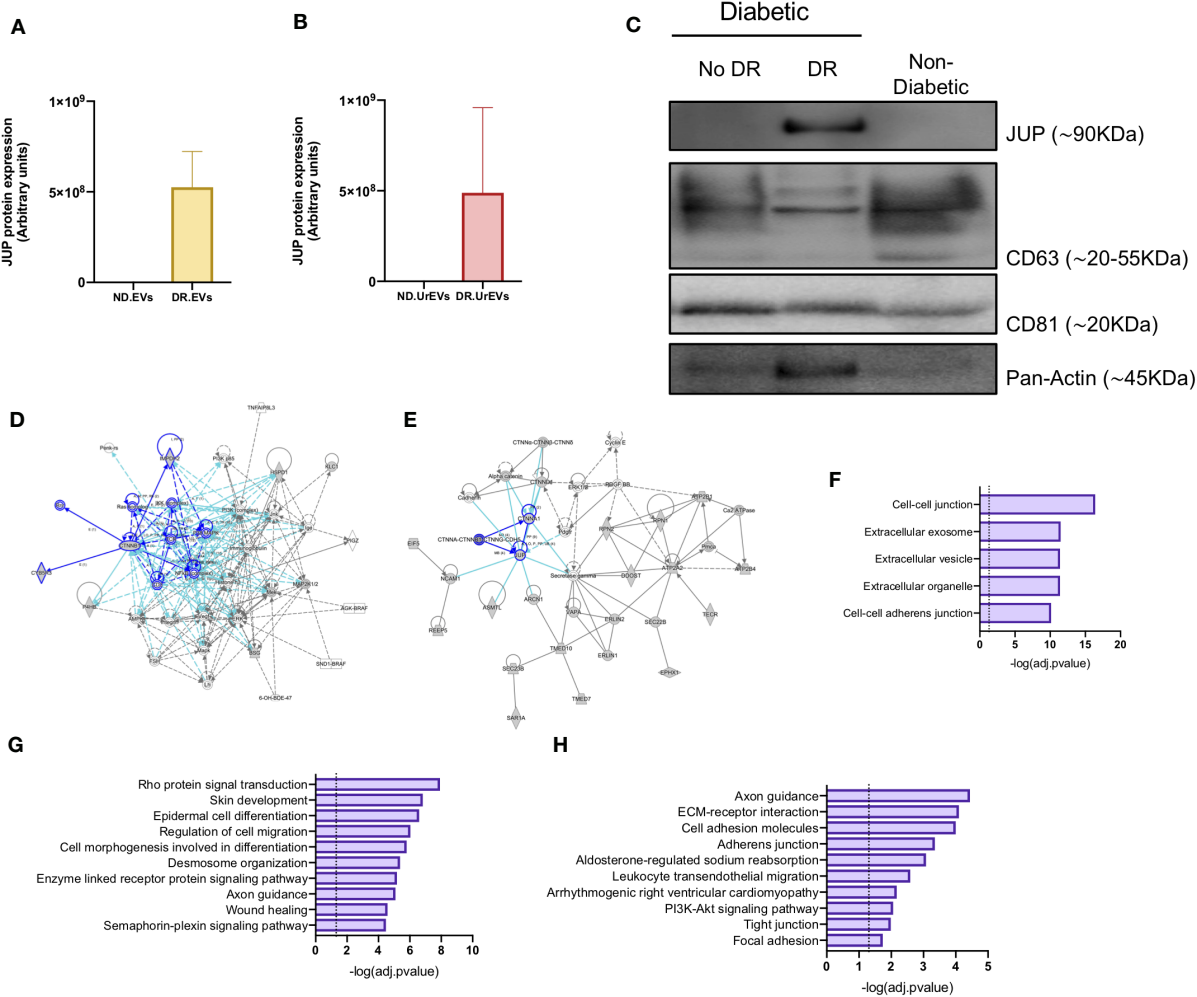
Validation of JUP as a potential biomarker for diabetic retinopathy. JUP protein levels in retinal EVs **(A)** and urinary EVs **(B)** from Non-diabetic and Diabetic patients, obtained from the proteomic datasets. **(C)** JUP, CD63, CD81 and Pan-Actin levels in urinary EVs pooled from Non-DR, DR and Non-Diabetic patients. Each lane contains 30ug of EV proteins obtained from a pool of three independent samples. A network diagram representing protein-protein interaction showing **(D)** proteins that are involved in the organization and disruption of anchoring junction and **(E)** proteins involved in the development of vasculature. **(F)** Prediction analysis to determine protein subcellular localization. Pathway **(G)** and biological process **(H)** enrichment analysis of shared proteins between DR.EVs and DR.UrEVs.

Pathway analysis performed on the 57 proteins detected in DR retinal tissue and urine-derived EVs showed a consistent representation of cell-to-cell junction-related proteins and pathways (**Figures 4C, D**). Among them, Junction plakoglobin (JUP) stands out with a strong association to DR due to its absence in control and healthy samples EV proteomic profiles (**Figures 5A, B**). To assess the potential of proteomics and integrative analysis in biomarker selection, we measured JUP protein (Gamma Catenin) expression in additional set of EVs. We assessed JUP expression in pooled retina-derived EV samples from diabetic patients with DR, diabetic patients without DR and non-diabetic patients, part of a different cohort from those used for proteomic analysis. Remarkably, JUP was only detected in EVs samples from DR patients and absent in EVs from both diabetic patients without DR and non-diabetic samples (**Figure 5C**). CD63 and CD8, EVs bona fide markers, were used as housekeeping controls. JUP network analysis revealed its role in cytoskeleton arrangement (**Figure 5D**), vascular integrity in endothelial cells (**Figure 5E**), and both critical processes in DR onset and progression (31). Finally, we performed pathway enrichment analysis using the Top 100 genes co-expressed with JUP identified with ARCHS4 RNA-seq gene-gene co-expression matrix (**Figures 5F–H**). JUP and co-expressed proteins were identified in cellular compartments such as cell-cell junction, extracellular vesicles, and cell-cell adherent junctions (**Figure 5F**). JUP is associated with pathways such as Rho protein signaling, cell migration, desmosome organization, wound healing, and others (**Figure 5G**). We also found associations with biological processes including cell adhesion, leukocyte trans-endothelial migration, and tight junction maintenance (**Figure 5H**).

## Discussion

Our study presents for the first time an integrative proteomic analysis of diabetic retinopathy tissue explants, retinal-derived EVs, and urinary EVs. Incidence of DR increases with the duration of the disease, particularly if accompanied by poor blood glucose control as evidenced by elevated glycated hemoglobin (A1C) levels, developing in more than 60% of type 2 and nearly all type 1 diabetic patients (1, 32) DR is identified in a third of people with diabetes and associated with increased risk of life-threatening systemic vascular complications, including stroke, coronary artery disease, and heart failure (31). DR causes 2.6% of vision loss worldwide (WHO, 2020), and these numbers are predicted to rise due to population growth, aging demographics, and the expanding diabetes epidemic worldwide. Alterations in the microvascular circulation of the retina lead to altered permeability, abnormal vascularization, production of inflammatory and oxidative stress mediators with resulting loss of vision (33). While state-of-the-art treatments, including photocoagulation and anti-Vascular Endothelial Growth Factor (VEGF) therapies (34, 35) are available to treat DR-induced retinopathy resulting in vision loss, diagnostic tools that provide early intervention and treatment to control or reverse disease are limited. The interest in EVs and their potential application as biomarkers across the spectrum of human diseases has increased since their first description more than 30 years ago (36, 37). Our study presents a distinct protein profile, exclusive to DR-derived EVs when compared to normal retinal tissue (**Figure 2E**). Next, we searched for proteins exclusively detected in urinary EVs from DR patients (**Figure 3F**). Despite emerging new data describing EVs isolated from neural retina (17), vitreous humor (38), aqueous humor (39), retinal astrocytes (40), and retinal pigment epithelium (41), our study is the first attempting to connect DR retinal and urinary EV proteomic profiles. Interestingly, Porta et al. recently described EVs in DR patient serum, containing upregulated miR-150-5p, miR-21-3p and miR-30b-5p, predicted to influence vessel formation, endothelial cell migration and vascular permeability (42). Our functional analysis of proteins detected in DR retinal tissue derived EVs showed a strong correlation to remodeling of epithelial adherent junctions, which is closely related to epithelial migration and permeability (43). Recent studies from Gurudas et al (44) proposed serum cystatin C as a biomarker for sight-threatening diabetic retinopathy, but they do not consider the potential advantages of EVs as robust biomarkers (21). JUP, also known as junction plakoglobin or gamma-catenin, is a member of the catenin protein family, a component of desmosomes and adherent junction structures (45). JUP forms complexes with β-catenin, directing actin filament formation and vesicle trafficking (46). During development, plakoglobin is localized to blood-retinal barrier microvessels (47)and it has been linked to cancer (48). Even more, JUP was detected in another set of pathology-associated EVs. Nuñez-Borque et al. identified JUP in anaphylactic reaction-derived EVs in human serum (14). Anaphylaxis, an extremely severe allergic reaction, is profoundly related to vascular permeability (49). Interestingly, JUP is involved in multiple pathogenic pathways related to DR. Our analysis presents JUP involved in leukocyte transendothelial migration and tight junction biology, supporting Porta et al’s (32) findings, and postulating endothelial cell dysfunction as a potential therapeutical target in DR. Junction plakoglobin levels are almost undetected in urinary EVs obtained from healthy donor samples and, more importantly in urinary EVs obtained from diabetic patients without diabetic retinopathy. Therefore, the detection of JUP-loaded EVs could be useful as a novel biomarker for DR using urinary EVs. Analysis of urinary DR.EVs showed that there are distinct EV proteomic profiles in DR patient urine vs non-diabetic urine, with changes relative to EV size, concentration and release or absorption rate. To our knowledge, no prior publication has studied changes in urinary EVs of DR patients. There is a connection between DR and diabetic nephropathy as they both involve microvascular pathologies created by uncontrolled and/or longstanding diabetes (50). In both conditions, pathologic alterations induced by hyperglycemia include NOS production, increased permeability, endothelial dysfunction, and activation of PKC and AGEs production (51). Bioinformatic comparison of protein signatures between diabetic and non-diabetic retina showed significant fold differences in biological processes associated with DR for diabetic retina, including EIF2 signaling, mitochondrial dysfunction, mTOR signaling, and oxidative phosphorylation, all widely characterized as involved in diabetes (52, 53). The use of urine EVs as a prognostic indicator for DR is reliant on the correlation of molecular signatures that are detected in retinal DR and urine DR EVs. Our analysis demonstrates this correlation. Furthermore, our data reveals a shared protein signature between retinal DR.EVs and urine DR.EVs, that potentially would allow for DR.urEV protein signatures to be used as DR prognostics. Our study also shows that the molecular cargo present in EVs from DR retina and urine samples have similarities which could lead to the development of a urine-based biomarker for DR and provide potential EV-based targets for drug intervention to reduce DR pathogenesis. DR diagnosis is complex. Further analysis of retinal and urine DR.EV differential expression and increased patient sample cohorts will greatly improve the development of JUP as an early screening biomarker for DR diagnosis with the potential of preventing disease progression and subsequent vision loss.

## Data availability statement

The data presented in the study are deposited in the ProteomeXchange Consortium via PRIDE repository, accession number PXD034626: https://www.ebi.ac.uk/pride/archive/.

## Ethics statement

The studies involving human participants were reviewed and approved by City University of New York, Lehman College (IRB). The patients/participants provided their written informed consent to participate in this study.

## Author contributions

JM and AR-N performed experiments, interpreted, and analyzed the data. CS, JZ, OO, SH and RG performed experiments. MF-B, LE interpreted data and reviewed the manuscript. DC designed patient data organization and analysis. AB-M and SR designed and supervised the work and wrote the manuscript. All authors contributed to the article and approved the submitted version.

## Supplementary material

Supplementary Figure 1 Histology of non-diabetic and diabetic retina. Representative sample image of non-diabetic retinal slice showing **(A)** phase light microscopy of the retina and **(B)** DAPI labeling (ex 358/em 461). nuclear staining of the lamina. Representative sample retinal slice showing diabetic retina showing **(C)** phase microscopy and **(D)** DAPI nuclear staining of the lamina. Scale: 100um.

Supplementary Table 1 List of proteins (264) exclusively detected in Diabetic retinopathy extracellular vesicles released from human retina tissue when compared with Nondiabetic EVs released by human retina tissue and the culture media.

Supplementary Table 2 List of proteins (364) exclusively detected in Diabetic retinopathy tissue explants from human retina tissue.

Supplementary Table 3 List of 228 proteins detected in Diabetic retinopathy extracellular vesicles when compared with diabetic retinopathy tissue explant secretome.

Supplementary Table 4 List of 1112 proteins detected in Diabetic retinopathy urinary extracellular vesicles, and not detected in non-diabetic urinary extracellular vesicles.
