# Supplementary figures and images for: Extracellular vesicles of human diabetic retinopathy retinal tissue and urine of diabetic retinopathy patients are enriched for the junction plakoglo bin protein
Source: Front Endocrinol (Lausanne). 2023 Jan 6;13:1077644. doi: 10.3389/fendo.2022.1077644 (PMC9854122; doi:10.3389/fendo.2022.1077644)

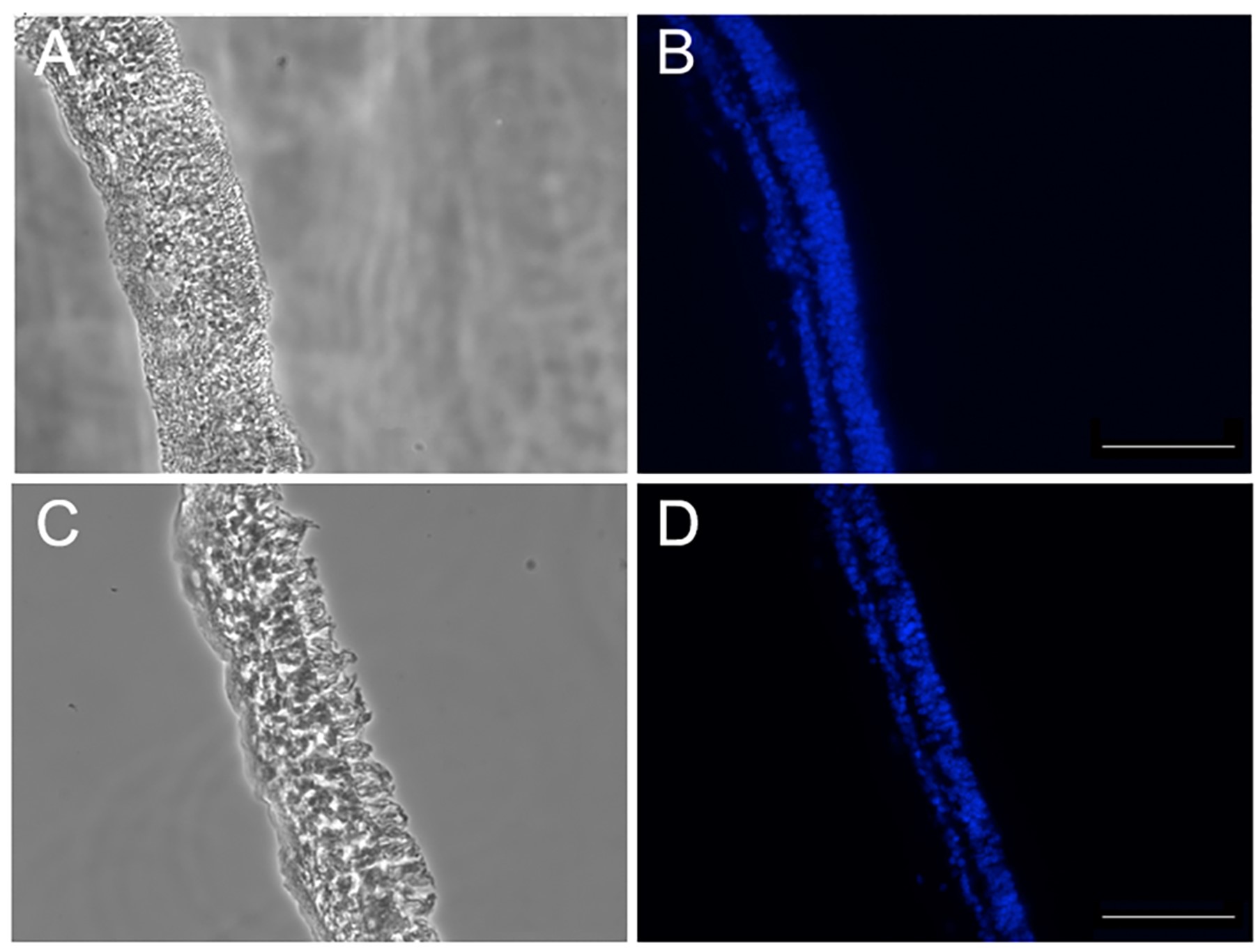

Supplement: Supplementary Figure 1 — Histology of non-diabetic and diabetic retina. Representative sample image of non-diabetic retinal slice showing (A) phase light microscopy of the retina and (B) DAPI labeling (ex 358/em 461). nuclear staining of the lamina. Representative sample retinal slice showing diabetic retina showing (C) phase microscopy and (D) DAPI nuclear staining of the lamina. Scale: 100um. [file Image_1.jpg]
